# Women’s Experiences With Maternity Care in Public and Private Healthcare Facilities in Western India: A Community-Based Cross-Sectional Study

**DOI:** 10.7759/cureus.81922

**Published:** 2025-04-08

**Authors:** Hiral Raval, Tapasvi Puwar, Nilam Patel, Apurvakumar Pandya, Aniket Rana, Bharti D Koria

**Affiliations:** 1 Public Health, Indian Institute of Public Health, Gandhinagar, IND; 2 Health and Family Welfare, Government of Gujarat, Gandhinagar, IND; 3 Community Medicine, Pujya Muktanandbapu Medical College and Research Institute, Junagadh, IND; 4 Community Medicine, Pandit Dindayal Upadhyay Medical College, Rajkot, IND

**Keywords:** childbirth experience, maternal care, maternal-child health, patient-provider interaction, respectful maternity care

## Abstract

Introduction: Quality improvement interventions for maternal healthcare services are often designed without input from women. Exploring women's childbirth experiences provides a unique understanding of care received in healthcare facilities. This study aimed to investigate women's childbirth experiences in Western India, with an objective to inform the development of woman-centered quality improvement interventions.

Methods: Data were collected from 186 postnatal women who delivered their babies at public or private healthcare facilities. A standardized tool, adapted from the respectful maternity care (RMC) charter, was used to assess women's experiences of care during childbirth.

Results: A high prevalence of disrespectful intrapartum care was observed. Ninety-seven percent of women reported experiencing at least one form of disrespect, with 61% experiencing three or more rights violations and 28% experiencing four or five violations. The study found that factors such as low maternal literacy (χ²=10.75, p<0.05), lower caste (χ²=10.19, p<0.05), and longer duration of stay at the health facility (χ²=8.30, p<0.05) were significantly associated with an increased risk of experiencing RMC violations. Other factors found to be statistically significant included marital status (χ²=5.35, p<0.05) and parity (χ²=6.67, p<0.05).

Conclusion: The study emphasizes the critical need for a multi-pronged approach to improve maternal healthcare in India. To ensure respectful and high-quality maternity care, possible measures include provider training to enhance competencies to be more responsive to women's needs, encouraging effective communication, strengthening system-level support, and empowering women to voice their needs during childbirth. Prospective research to understand local contextual elements, societal norms, and patient-provider communication would be valuable.

## Introduction

Globally, maternal mortality is widely acknowledged as a key indicator of health system effectiveness as well as a reflection of social development and equity. India has committed to the United Nations' Sustainable Development Goal of reducing Maternal Mortality Ratio (MMR) to 70 per 1,000 live births by 2030 [1]. The Registrar General of India's Sample Registration System (RGI-SRS) report observes a steady decline in MMR, showing that it was 130 (2014-16), 122 (2015-17), 113 (2016-18), 103 (2017-19), and 97 (2018-20) [2]. Such a steady decline in MMR is the result of emphasizing WHO’s four key strategies for MMR reduction: provisioning quality of care; demand-side financing (e.g., Janani Shishu Suraksha Karyakaram (JSSK), Janani Suraksha Yojana (JSY)); addressing social determinants of maternal health; and public-private partnerships [3,4]. Although India's MMR has decreased, this progress must be assessed by examining both access to and quality of maternal health services. Increased access is necessary, but the quality of care dictates whether it translates to better health outcomes. In this direction, India has implemented several initiatives to improve the quality of maternal care. Pradhan Mantri Surakshit Matritva Abhiyan (PMSMA), Surakshit Matrutva Ashvashan (SMA), the Labour Room and Maternity Quality Improvement Initiative (LaQshya), and Maternal and Perinatal Death Surveillance and Response (MPDSR) are examples of a few initiatives India has put in place to enhance emergency obstetric care services, increase access to prenatal, intrapartum, and postnatal care, and raise the standard of care given in medical facilities [4].

The WHO highlights the significance of providing women and children with high-quality care, emphasizing continuum care as a key component in lowering avoidable maternal and infant mortality [5]. This requires a well-coordinated approach to maternal care during pregnancy, childbirth, and the postpartum period. Significant progress has been achieved in enhancing MMR through infrastructure improvement, availability of human resources, transportation services [6,7], training, and provision of incentives, but there are still challenges that command our urgent attention.

Despite notable efforts, instances of negligence, abuse, and humiliation of women during childbirth continue to be major obstacles to high-quality care in India and around the world [8,9]. A study conducted in rural India reported that 40% of women experienced some form of disrespect or abuse during childbirth [10]. The authors' previous study revealed noncompliance with respectful maternity care (RMC) standards in public health facilities right from primary, secondary, and tertiary care services [11]. These experiences can have adverse impacts on their physical and mental health, which influence future healthcare-seeking behaviors [12,13]. Women might experience profound psychological distress from experiences of receiving maternal care, which could undermine their trust in the healthcare system and discourage them from seeking care. In order to provide respectful maternity care, which is a fundamental human right and a prerequisite for attaining high-quality maternal care, it is imperative to address these challenges.

The humane and dignified treatment of women during pregnancy, childbirth, and the postpartum period is known as respectful maternity care (RMC) [14]. It promotes the use of encouraging words and demonstrates positive attitudes while placing women's rights and their preferences as a top priority during service delivery [15]. Fundamental values of RMC delivery include informed consent, respect and dignity, confidentiality, empowerment, and freedom from abuse and violence [16].

This study attempts to understand women's experiences of receiving maternity care in both public and private healthcare facilities in Western India. The primary objectives of the study are to identify instances of violations of respectful maternity care standards and recognize factors contributing to compromised quality of care. The findings of the study will be utilized to inform the development of strategies for ensuring respectful and dignified care during pregnancy, childbirth, and postpartum periods.

## Materials and methods

Study design, population, and study duration

This study was planned and executed at the Indian Institute of Public Health, Gandhinagar, Gujarat. The cross-sectional study design was adopted for the study. Postpartum women of any age who delivered at a public or private healthcare facility in the past two months were interviewed during December 2019 and January 2020.

Study setting

The study was conducted in the Gandhinagar district of Gujarat State, India [10]. Out of four blocks of Gandhinagar, we have selected one block randomly using the chit-pick method. The selected block had eight primary health centers (PHCs), and we had randomly chosen three PHCs again using the chit-pick method. Postpartum women received maternal care in both public and private healthcare facilities. Around three PHC areas within a selected block were approached for the study.

To ensure unbiased and reliable sampling using the chit-pick method, the four blocks of Gandhinagar were first defined as the sampling frame. By creating standardized chits (using the same type of white paper, thickness: 75 grams per square meter (GSM), and texture), folding them in the same way (i.e., twice over), and hiding them in a non-transparent container (cloth bag), equal probability was maintained. In accordance with the documented protocol, a blindfolded researcher (first author) performed the draw in the presence of a senior researcher (second author). In order to ensure reproducibility and transparency, thorough documentation of the procedure was kept, including the date, time, location, names of those participating in the draw, etc. Researchers ensured ethical considerations by maintaining fairness and informed participation. Moreover, the selected block was compared with the other three blocks for geographic and service coverage in order to assess the representativeness as part of post-hoc validation. The same principle was applied for the selection of PHC areas.

Sample size

A total of 186 postpartum women were recruited for the study. The authors identified 350 deliveries that occurred at the health facilities within the two months preceding data collection, with 204 (58%) in public and 146 (42%) in private facilities. A 50% representative sample of 175 potential participants was drawn from this list using systematic sampling, where every second name was selected. Accounting for a 10% non-response rate (i.e., 17 individuals), the sample size calculated was 192. However, four women migrated, and two declined to participate in the study, resulting in a final sample size of 186. This sample remained representative, as the proportions of deliveries occurring in public and private facilities (59% and 41%, respectively) closely matched the proportions observed in the total recorded deliveries.

Inclusion and exclusion criteria

Inclusion criteria for the study included women who had delivered at the health facility within the past two months, were between 18 and 45 years old, and provided informed consent to participate. Exclusion criteria included women who were unavailable for interviews during the study visit and those who had migrated from the study area.

Data collection tool

A standardized study tool was developed based on the RMC Care Standard US Agency for International Development (USAID) tool, with modifications to include necessary demographic data [11]. This tool comprised 49 questions encompassing sociodemographic characteristics (questions 1-12), maternal characteristics (questions 13-15), and seven RMC parameters (16-49). These RMC parameters were physical harm or ill-treatment (right to freedom from harm and ill-treatment); non-consented care (right to information, informed consent, and choice, including companionship during maternity care); non-confidentiality care (right to confidentiality and privacy); non-dignified care (right to dignity and respect); discrimination based on specific attributes (right to equality and equitable care without discrimination); abandonment or denial of care (right to timely healthcare and to the highest attainable level of health); and detention or confinement in facilities (right to liberty, autonomy, self-determination, and freedom from coercion). A pilot study was conducted with 10 women to assess the feasibility and practicality of the study pro forma and design. Based on the findings of the pilot study, modifications were made to the data collection tool.

Data were collected through home visits to the selected participants. Interviews were conducted with eligible women who were available and provided informed consent after explaining the details of the research. For women who were unavailable or untraceable, follow-up attempts were made during the MAMTA sessions at the respective Anganwadi Centres (Child Care Centre). At these sessions, women were informed about the study, and those who provided consent for voluntary participation were thereafter included in the study and interviewed.

Data analysis

Data were entered into Microsoft Excel (Microsoft Corporation, Redmond, Washington, United States) and subsequently analyzed using Epi Info 7.0 (Centers for Disease Control and Prevention (CDC), Atlanta, Georgia (US)). Data entry checks and codes were implemented to ensure data completeness. Missing data were addressed by contacting participants. Records with persistently missing data were excluded from the final analysis. Descriptive statistics were used to summarize sample demographics and key aspects of care. Chi-square tests were applied to explore associations between socio-demographic factors and reported outcomes, with a significance level set at 0.05.

Ethical considerations

Administrative approval of the study was obtained from the Family and Welfare Department, Government of Gujarat, whereas ethical approval was received from the Institutional Ethics Committee of the Indian Institute of Public Health Gandhinagar (IIPHG), Gujarat, India. Prior to participation, all participants were provided with detailed information about the study's objectives and procedures in their local language. Informed consent was obtained from each participant, and the confidentiality of their personal information was strictly maintained.

## Results

Socio-demographic characteristics of the study participants

Of the 186 women interviewed, 49% (n=92) were aged between 21 and 25 years. Regarding education, 65% (n=121) had completed higher secondary education, while only 8% (n=15) had attained a higher level of education. Notably, 16% (n=29) of the women were illiterate. About 89% (n=165) of postpartum women were homemakers. It was noted that 59% (n=110) of the deliveries occurred in government facilities, and the rest, about 41% (n=76), of the deliveries took place in private healthcare facilities. A total of 64% (n=119) of deliveries were vaginal. The majority of participants (68%, n=126) used private vehicles to reach the hospital. Half of the participants (51%, n=95) delivered a girl child. Table 1 presents the sociodemographic characteristics of the study participants.

**Table 1:** Sociodemographic characteristics of study participants (N=186)

Characteristics	f	%
Mother’s Age	-	-
Up to 20 years	30	16%
21 to 25 years	92	49%
26 to 30 years	48	26%
30 to 45 years	16	9%
Mother Literacy	-	-
Illiterate	29	16%
Primary education	21	11%
Secondary education	121	65%
Higher secondary and above	15	8%
Mother's Occupation	-	-
Housewife	165	89%
Agriculture/labor	19	10%
Employee (government or private)	2	1%
Place of Delivery	-	-
Private healthcare facilities	76	41%
Public healthcare facilities	110	59%
Parity	-	-
Primipara	79	42%
Multipara	107	58%
Type of Delivery	-	-
Normal vaginal delivery	119	64%
Cesarean section delivery	67	36%
Birth Outcomes	-	-
Alive girl child	95	51%
Alive boy child	87	47%
Alive twins (any)	2	1%
Stillbirth	2	1 %

Table 2 presents the results of different types of disrespect experienced by participants according to the RMC charter rights violated. Charter right no. 2 was most commonly reported (94%, n=175) to be violated, followed by charter rights no. 6 and 1, 69% (n=129 and n=128) each, respectively.

**Table 2:** Respectful maternity care charterwise prevalence of rights violated (N=186)

Charter right No	Charter right	Rights violated *f(%)*	Rights not violated *f(%)*
1	Physical harm and ill-treatment	128(68.8)	58(31.2)
2	Right to information, informed consent, and preferred choice	175(94.1)	119(5.9)
3	Confidentiality and privacy	98(52.7)	88(47.3)
4	Dignity and respect	41(22)	145(78)
5	Provision of equitable care, free of discrimination	14(7.5)	172(92.5)
6	Left without care	129(69.4)	57(30.6)
7	Detained or confined against one's will	11(5.9)	175(94.1)

Table 3 depicts the prevalence of rights violations experienced by patients during childbirth. A small percentage (2.7%, n=5) of participants reported no violations of their rights. Consequently, the overall prevalence of rights violations across all types of healthcare facilities was 97.3% (n=181). Notably, more than half (59%, n=110) of participants reported violations of at least three rights outlined in the RMC charter standards.

**Table 3:** Overall prevalence of violations of the respectful maternity care charter (N=186)

Participants responses	Any rights violated *f(%)*
Never abused/No violation	5(2.7%)
Any three rights violated	110(59.1%)
Any four to five rights violated	59(31.7%)
Any six to seven rights violated	12(6.5%)

Factors associated with rights violation 

Analysis revealed several sociodemographic and obstetric factors associated with violations of RMC standards. Table 4 presents the statistical findings of the study. Statistically significant associations were observed with maternal literacy status (χ²=10.75, df=3, N=186, p<0.05), caste (χ²=10.19, df=4, N=186, p<0.05), marital status (χ²=5.35, df=1, N=186, p<0.05), parity (χ²=6.67, df=1, N=186, p<0.05), duration of stay at the health facility (χ²=8.30, df=2, N=186, p<0.05), and duration of labor in the labor room (χ²=10.8, df=1, N=186, p<0.05). In contrast, no significant associations were found between RMC violations and maternal age, occupation, socioeconomic status, presence of an accompanying person during delivery, the occurrence of delivery complications, or the number of antenatal care (ANC) visits.

**Table 4:** Factors associated with violation of respectful maternity care charter (N=186) *Statistical significance was determined using a significance level of 0.05. SC: schedule caste; ST: schedule tribe; OBC: other backward class; BPL: below poverty line; RMC: respectful maternity care; ANC: antenatal care

Variables	Violation of any of the RMC sub-items			Total	Chi-square value
Mother's Age at Time of Delivery	Yes	No	-		0.69
Up to 20 years	21(11.3)	09(4.8)	30(16.1)		
21 to 25 years	67(36.0)	25(13.4)	92(49.5)		
26 to 30 years	37(19.9)	11(5.9)	48(25.8)		
More than 30 years	11(5.9)	5(2.7)	16(8.6)		
Mother's Literacy	-	-	-		10.75*
Illiterate	21(11.3)	08(4.3)	29(15.6)		
Primary education	11(5.9)	10(5.4)	21(11.3)		
Secondary education	97(52.2)	24(12.9)	121(65.1)		
Higher education	08(4.3)	07(3.8)	15(8.1)		
Mother's Occupation	-	-	-		2.06
Housewife	119(64.0)	46(24.7)	165(88.7)		
Agriculture/daily wage labour	11(5.9)	08(4.3)	19(10.2)		
Employee	1(0.5)	1(0.5)	2(1.1)		
Mothers Caste	-	-	-		10.19*
General	07(3.8)	08(4.3)	15(8.1)		
SC	08(4.3)	07(3.8)	15(8.1)		
ST	04(2.2)	01(0.5)	5(2.7)		
OBC	109(58.6)	33(17.7)	142(76.3)		
Don’t want to declare	05(2.7)	04(2.2)	09(4.8)		
BPL Family	-	-	-		0.0018
Yes	86(46.2)	18(9.7)	104(55.9)		
No	68(36.5)	14(7.5)	82(44.0)		
Marital Status	-	-	-		5.35*
Ever married	131(70.4)	28(15.1)	159(85.5)		
Others	17(9.1)	10(5.4)	27(14.5)		
Family Attendant Present	-	-	-		-
Husband	38(20.4)	38(20.4)	76(40.9)		0.511
In-laws	34(18.3)	40(21.5)	74(39.8)		
Others	19(10.2)	17(9.1)	36(19.4)		
Parity	-	-	-		6.67*
Primipara	44(23.7)	35(18.8)	79(42.5)		
Multipara	79(42.5)	28(15.1)	107(57.5)		
ANC Registration and Visits	-	-	-		0.30
< 2	15(8.1)	16(5.9)	31(14.0)		
2-4	22(11.8)	20(8.1)	42(19.9)		
>4	61(32.8)	52(28)	113(50.0)		
Stay at a Health Facility	-	-	-		8.30*
<12 hrs	17(9.1)	15(8.1)	32(17.2)		
12-24 hrs	09(4.8)	08(4.3)	17(9.1)		
>24 hrs	103(55.4)	4(18.3)	137(73.7)		
Delivery With Complications	-	-	-		3.16
Yes	03(1.6)	08(4.3)	13(7.0)		
No	96(51.6)	79(42.5)	173(93.0)		
Waiting Time in the Labor Room	-	-	-		10.8*
<6 hrs	77(41.4)	25(13.4)	102(54.8)		
>6 hrs	44(23.7)	40(21.5)	84(45.2)		

A chi-square test for independent variables was applied to see the association between the variables like types of health care facility (X²=17.96, df= 1, N=186, P<0.05), time of delivering baby (X²=6.11, df= 1, N=186, P<0.05), type of delivery attendant (X²=24.20, df= 2, N=186, P<0.05), mode of reaching hospital (X²=29.42, df= 2, N=186, P<0.05), and gender of delivering person (X²=12.62, df= 1, N=186, P<0.05) were found to have a significant association with violation of RMC standards. No association was found between mode of delivery, outcome of delivery, and violation of standards.

## Discussion

Our study found that 97% (n=180) of participants experienced at least one violation of their RMC rights, indicating widespread instances of abuse and disrespect during intrapartum care. This finding aligns with a study conducted in North Showa, Ethiopia, where 51.4% of institutional deliveries reported experiencing some form of abuse or disrespect [16]. Numerous studies worldwide have investigated factors contributing to violations of RMC standards, which can originate from both the provider and the patient side [12,13]. Identifying and addressing these predictive factors remains crucial for improving the quality of maternity care.

The most frequently violated RMC standard in our study was the right to information, informed consent, and protection of choices and preferences (94%, n=175), mirroring findings from an Ethiopian study (48.9% non-consented care) [6] and an Indian study (62.26% non-consented care) [17]. This highlights the critical need to prioritize informed consent and respect for women's choices as fundamental aspects of maternity care.

Overall, 97% (n=180) of women in our study reported experiencing some form of disrespect or abuse, encompassing violations such as lack of information and informed consent (94%, n=175), being left without care after delivery (69%, n=129), experiencing physical harm (69%, n=128), and lacking privacy and confidentiality (53%, n=98). These findings are consistent with those of other studies that have reported varying proportions of RMC standard violations during childbirth [6,16-19].

The WHO has outlined 30 intrapartum standards for respectful maternity care [20], providing evidence-based guidance for ensuring quality care. Our study identified several factors associated with increased RMC violations, including maternal literacy status, caste, marital status, parity, duration of stay at the health facility, and duration of labor in the labor room. These findings are supported by other studies that have identified various demographic, socioeconomic, and provider-level factors influencing the quality of maternity care [12,16-19].

A qualitative study in Tehran, Iran, identified four key influencing factors: individual-level factors (e.g., provider perceptions of women's limited knowledge), healthcare provider-level factors (e.g., provider stress, workload), hospital-level factors (e.g., staff shortages), and national health system-level factors [21]. Conversely, having a companion during delivery, higher education, and prior ANC visits were identified as protective factors in our study, suggesting the positive influence of social support and access to healthcare services [22]. A qualitative evidence synthesis analyzing studies from 22 countries found that partners played crucial roles in supporting women during childbirth, including providing information, reviewing communication with staff, offering emotional support, and assisting during labor [23]. Partner support has also been associated with positive birth outcomes [22].

Implications for research, practice, and policy

All healthcare providers involved in maternity care must receive comprehensive training and awareness-raising programs on RMC principles, including respectful communication, informed consent, and the importance of addressing women's needs and preferences. High-quality maternity care necessitates robust governance, a motivated and well-supported healthcare workforce, adequate financing, updated reporting systems, appropriate technological assistance, and a well-maintained logistical chain [21,23,24]. 

Promoting early and regular antenatal care can empower women to communicate effectively with healthcare providers and seek timely care during emergencies. Public awareness campaigns should be conducted through media, newspapers, and community workers to educate women about their rights to respectful maternity care [24,25]. Continuous training, retraining, and refresher courses should be implemented to reinforce respectful behaviors and communication among healthcare providers.

Strengths and limitations of the study

This community-based cross-sectional study offers valuable insights into women's experiences of maternity care in a natural program setting. Conducting interviews in women's homes likely fostered a more open and honest dialogue. Furthermore, focusing on women who had recently given birth in the last two months may have minimized recall bias. While acknowledging these strengths, the authors acknowledge certain limitations. The relatively small sample size and the study's limited duration may have constrained the scope of findings and limited the generalizability of the results. The reliance on self-reported data may have introduced some level of recall bias. Moreover, the study has not comprehensively explored all potential factors influencing RMC, including provider stress, workload, system-level barriers, and consumer-side behaviors. Future research should delve deeper into the complex interplay of factors influencing RMC violations. Furthermore, research investigating the impact of interventions aimed at improving RMC, such as training programs, quality improvement initiatives, and patient safety programs, is crucial for informing future policies and improving the quality of maternity care.

## Conclusions

The violations of respectful maternity care standards underscore the need for multi-pronged actions to enhance the quality of maternal healthcare service delivery in India. Creating a supportive environment for women and eliminating maltreatment at healthcare facilities remain essential concerns. To ensure that all women have access to respectful and high-quality maternity care, measures such as improving provider training, promoting effective communication, enhancing system-level support, and empowering women to advocate for their rights are warranted.

## References

[1] (2025). Maternal and Adolescent Healthcare. https://mohfw.gov.in/sites/default/files/3201617.pdf.

[2] (2025). Significant Decline in the Maternal Mortality Ratio (MMR) From 130 in 2014-16 to 97 per Lakh Live Births in 2018-20: Dr. Mansukh Mandaviya. https://pib.gov.in/PressReleaseIframePage.aspx?PRID=1879912.

[3] Singh Singh, P.S P.S (2018). India Has Achieved Ground-Breaking Success in Reducing Maternal Mortality. https://www.who.int/southeastasia/news/detail/10-06-2018-india-has-achieved-groundbreaking-success-in-reducing-maternal-mortality.

[4] (2024). Quality of Care in India. https://www.qualityofcarenetwork.org/country-data/india.

[5] World Health Organization (2024). World Health Organization. Promoting Respectful Maternity Care: A Resource Package. Respectful Maternity Care Orientation Package.

[6] (2024). Cabinet Apprised of Achievements Under National Health Mission (2021-24): A milestone in improving India’s public health outcomes. https://pib.gov.in/PressReleasePage.aspx?PRID=2095055#:~:text=India%20has%20.

[7] (2014). Maternal Health Programme. https://mohfw.gov.in/sites/default/files/Chapter415.pdf.

[8] Bohren MA, Vogel JP, Hunter EC (2015). The mistreatment of women during childbirth in health facilities globally: a mixed-methods systematic review. PLoS Med.

[9] (2024). Women Still Face Abuse During Childbirth at Health Facilities. https://timesofindia.indiatimes.com/city/patna/women-still-face-abuse-during-childbirth-at-health-facilities/articleshow/100560163.cms.

[10] Sharma G, Penn-Kekana L, Halder K, Filippi V (2019). An investigation into mistreatment of women during labour and childbirth in maternity care facilities in Uttar Pradesh, India: a mixed methods study. Reprod Health.

[11] Raval H, Puwar T, Vaghela P, Mankiwala M, Pandya AK, Kotwani P (2021). Respectful maternity care in public health care facilities in Gujarat: a direct observation study. J Family Med Prim Care.

[12] Hosseini Tabar J, Shahoie R, Zaheri F, Mansori K, Hashemi Nasab L (2023). Prevalence of disrespect and abuse during childbirth and its related factors in women hospitalized in the postpartum ward. J Family Med Prim Care.

[13] Afulani PA, Kelly AM, Buback L, Asunka J, Kirumbi L, Lyndon A (2020). Providers' perceptions of disrespect and abuse during childbirth: a mixed-methods study in Kenya. Health Policy Plan.

[14] (2024). Respectful Maternity Care (RMC) Charter. https://content.sph.harvard.edu/wwwhsph/sites/2413/2014/05/Final_RMC_Charter.pdf.

[15] Hill K, Stalls S, Sethi R, Jhpiego EB, Moffson S (2024). Moving Respectful Maternity Care into Practice in Comprehensive MCSP Maternal and Newborn Programs Operational Guidance. Moving Respectful Maternity Care into Practice in Comprehensive MCSP Maternal and Newborn Programs Operational Guidance.

[16] Amare NS, Mekuriyaw AM, Tesema GW, Ambaw YL (2022). Proportion and associated factors of respectful maternity care during childbirth in North Showa zone public health institutions, North Showa, Ethiopia: an institutional-based cross-sectional study. Front Public Health.

[17] Usso AA, Adem HA, Alemu A, Mohammed A (2023). Disrespect and abuse during childbirth in East Hararghe Zone public health facilities, eastern Ethiopia: a cross-sectional study. Front Glob Womens Health.

[18] Dynes MM, Twentyman E, Kelly L (2018). Patient and provider determinants for receipt of three dimensions of respectful maternity care in Kigoma Region, Tanzania-April-July, 2016. Reprod Health.

[19] Yadav A, Singh TB, Sachan S (2022). Prevalence of disrespect and abuse and its determinants during antenatal care services in rural Uttar Pradesh India. Int J Health Sci Res.

[20] World Health Organization (2018). Evidence and Recommendations. WHO Recommendations: Intrapartum Care for a Positive Childbirth Experience.

[21] Mirzania M, Shakibazadeh E, Bohren MA, Hantoushzadeh S, Babaey F, Khajavi A, Foroushani AR (2023). Mistreatment of women during childbirth and its influencing factors in public maternity hospitals in Tehran, Iran: a multi-stakeholder qualitative study. Reprod Health.

[22] (2024). Why Having a Companion During Labour and Childbirth May Be Better for You?. https://www.who.int/news/item/19-03-2019-why-having-a-companion-during-labour-and-childbirth-may-be-better-for-you.

[23] Jungari S, Sharma B, Wagh D (2021). Beyond maternal mortality: a systematic review of evidences on mistreatment and disrespect during childbirth in health facilities in India. Trauma Violence Abuse.

[24] Rajbangshi PR, Srivastava A, Nambiar D (2022). Women's experiences with maternity care in public health facilities of Assam, India. WHO South East Asia J Public Health.

[25] Yadav P, Smitha MV, Jacob J, Begum J (2022). Intrapartum respectful maternity care practices and its barriers in Eastern India. J Family Med Prim Care.

